# What the F‐POD? Comparing the F‐POD and C‐POD for monitoring of harbor porpoise (*Phocoena phocoena*)

**DOI:** 10.1002/ece3.10186

**Published:** 2023-06-09

**Authors:** Nicole Rose Eileen Todd, Ailbhe Sarah Kavanagh, Emer Rogan, Mark John Jessopp

**Affiliations:** ^1^ MaREI Centre Environmental Research Institute, University College Cork Cork Ireland; ^2^ School of Biological, Earth & Environmental Sciences (BEES) University College Cork Cork Ireland; ^3^ Fisheries Ecosystems Advisory Services (FEAS) Marine Institute Oranmore Ireland

**Keywords:** cetaceans, click detector, echolocation, foraging rate, long‐term monitoring programs

## Abstract

Passive acoustic monitoring (PAM) is a cost‐effective method for monitoring cetacean populations compared with techniques such as aerial and ship‐based surveys. The Cetacean POrpoise Detector (C‐POD) has become an integral tool in monitoring programs globally for over a decade, providing standardized metrics of occurrence that can be compared across time and space. However, the phasing out of C‐PODs following the development of the new Full waveform capture POD (F‐POD) with increased sensitivity, improved train detection, and reduced false‐positive rates represents an important methodological change in data collection, particularly when being introduced into existing monitoring programs. Here, we compare the performance of the C‐POD with that of its successor, the F‐POD, co‐deployed in a field setting for 15 months, to monitor harbor porpoise (*Phocoena phocoena*). While similar temporal trends in detections were found for both devices, the C‐POD detected only 58% of the detection‐positive minutes (DPM), recorded by the F‐POD. Differences in detection rates were not consistent through time making it difficult to apply a correction factor or directly compare results obtained from the two PODs. Generalized additive models (GAMs) were used to test whether these differences in detection rates would have an effect on analyses of temporal patterns and environmental drivers of occurrence. No differences were found in seasonal patterns or the environmental correlates of porpoise occurrence (month, diel period, temperature, environmental noise, and tide). However, the C‐POD failed to detect sufficient foraging rates to identify temporal patterns in foraging behavior, which were shown by the F‐POD. Our results suggest that the switch to F‐PODs will have little effect on determining broad‐scale seasonal patterns of occurrence but may improve our understanding of fine‐scale behaviors such as foraging. We highlight how care must be taken interpreting F‐POD results as indicative of increased occurrence when used in time‐series analysis.

## Graphical abstract

Static acoustic monitoring using POrpoise Detectors (PODs) has been an integral part of cetacean monitoring programs exploring habitat use and behavior. This study has given insights that the Full waveform capture POD will be invaluable for future monitoring of harbor porpoise and other cetacean species; however, care and consideration must be taken to make Cetacean‐POD data adaptable for the integration into future studies.

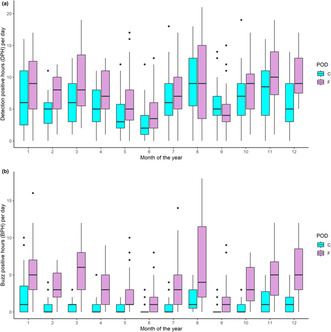

## 1 INTRODUCTION

Passive acoustic monitoring (PAM) is a well‐established method used to monitor acoustically active species and the habitat they reside in (Merchant et al., 2015). Over the years PAM technology has greatly advanced the field of cetacean ecology, allowing a cost‐effective alternative to extensive visual surveys that are not reliant on daylight hours, favorable weather conditions, and availability of observers. Passive acoustic monitoring of cetaceans has resulted in temporally high‐resolution data providing us with insights into population size and abundance (Amundin et al., 2022; Marques et al., 2013), habitat use (Fleming et al., 2018; Palmer et al., 2019), and behavior (Malinka et al., 2021; Pirotta, Thompson, et al., 2014; Todd et al., 2022) for many species. Such technology is also fundamental for long‐term monitoring, particularly with the increase in coastal developments and potential disturbance from construction, marine renewable devices, shipping, and fisheries (e.g., Fernandez‐Betelu et al., 2022; Omeyer et al., 2020; Ramesh et al., 2021; Todd et al., 2020, 2022).

While there are many useful applications of PAM, fixed autonomous acoustic recording devices can increase deployment times and sampling frequencies (Sousa‐Lima et al., 2013). Data loggers or echolocation click detectors, such as the Cetacean POrpoise Detector (C‐POD) (Chelonia Ltd., 2022) are a user‐friendly, relatively inexpensive device that can be deployed for continuous monitoring periods of 3–6 months. C‐PODs detect individual echolocation clicks between 20 and 160 kHz and have been a popular tool used to study odontocete ecology and behavior worldwide (e.g., Carstensen et al., 2006; Garagouni, 2019; Jaramillo‐Legorreta et al., 2017; Nykänen, 2016; Simon et al., 2010). Although no waveform data are stored by the devices, summary data on each click are preserved allowing post‐deployment classification of detected sounds into sequences called click trains. Further data analysis is then performed where click trains are assigned to dolphin or porpoise origins based on frequency and bandwidth. While it is often not possible to differentiate between dolphin species (Robbins et al., 2015), based on in‐field testing, Roberts and Read (2015) reported that C‐PODs perform well with a relatively high accuracy in detecting cetacean echolocation. C‐PODs have been used for over a decade and now form the basis of valuable long‐term monitoring datasets. The Full waveform capture PODs (F‐PODs) are the successor of the C‐PODs, and the manufacturer is recommending a transition from C‐PODs to F‐PODs as availability and support for C‐PODs may be limited in the coming years. This may have important implications for long‐term monitoring programs (and the associated archival data from such) as C‐PODs are replaced due to equipment loss (often as a result of storms, theft, etc.) or reach the end of their operational lifetimes. The F‐PODs have been designed to improve and upgrade the data associated with C‐PODs by recording more details of selected clicks including position of loudest cycle, frequency range, and capture of full waveform (Chelonia Ltd., 2022). These new features enhance train detection, providing increased sensitivity (within an average detection range of 400 m (Amundin et al., 2022; Nuuttila et al., 2018), assuming comparability with their C‐POD counterparts) with lower false positive rates compared with C‐PODs (Chelonia Ltd., 2022).

One of the main advantages of PAM is its potential to be implemented in long‐term monitoring programs to study the change in species occurrence and behavior over longer temporal scales. Many studies currently using C‐PODs need to ensure the longevity of their data for monitoring purposes, particularly in the light of climate change and habitat alterations through coastal developments. However, to date, there have been no studies reporting how the C‐POD and its successor the F‐POD compare in detection capacity and ability to identify trends in spatiotemporal drivers of detected cetaceans. In this study, we used data from a co‐deployed C‐POD and F‐POD to compare the performance of the PODs in detecting habour porpoise (*Phocoena phocoena*) across various commonly used detection metrics. Additionally, Generalized additive models (GAMs) were used to explore how both PODs identified spatiotemporal variation in harbor porpoise occurrence and foraging activity in relation to environmental variables.

## 2 METHODS

### 2.1 Data collection

Between April 2021 and July 2022 click detectors were deployed off Sherkin Island in Roaringwater Bay (51°27′40.7”N, 9°26′24.7”W), a Special Area of Conservation (SAC) for harbor porpoise, with an approximate area of 142.6 km^2^. Porpoise occur in the bay throughout the year, peaking in autumn months, with an estimated population (in 2008) of 117–201 individuals (NPWS, 2014). Density estimates have reportedly declined in recent years with the 2020 estimate of 0.61 individuals per km^2^ (O'Brien & Berrow, 2020), compared with the previous 2015 estimate of 2.02 individuals per km^2^ (O'Brien & Berrow, 2015). The deployment site is a relatively sheltered site on the southwest coast of Sherkin Island, in approximately 18 m water depth, with a predominantly sandy seafloor. One C‐POD and one F‐POD were co‐deployed on a mooring line anchored to the seabed, positioned 5 m above the seabed, side by side in a custom‐built acetal plastic frame to optimize simultaneous detections on both devices (Online Appendix, Figure S1). The devices were retrieved and redeployed every 3–4 months to ensure the continuity of acoustic recordings. Both the C‐POD and the F‐POD were calibrated (standardized) by Chelonia prior to the experiment, following their standard procedures (Chelonia Ltd., 2022).

### 2.2 Data analysis

C‐POD data were processed using Chelonia CPOD.exe software (V. 2044) and an inbuilt KERNO classifier to detect harbor porpoise click trains. F‐POD data were processed in a similar manner using the custom F‐POD.exe software (V 1.1) and KERNO‐F classifier (Chelonia Ltd., 2022). Click trains were classified as “NBHF” (narrowband high frequency) and all train quality classes were exported for further examination. Train quality filters are defined as “Hi” (high), “Mod” (moderate), and “Lo” (low). Detections were visually verified following guidelines from the manufacturer (Chelonia Ltd., 2022). This involved evaluating a subset of detection‐positive minutes (DPM) (100 random sampling points are recommended) for “Hi” and “Mod” trains, while all “Lo” trains were inspected as greater instances of false positives are sometimes reported for these trains (Clausen et al., 2018). Data were exported as different detection metrics: number of clicks (NClx), detection‐positive days (DPD), detection‐positive hours (DPH), and DPM.

Detection metrics were summarized for each deployment across three groupings of train quality filters, specifically HiModLo, HiMod, and Hi, reflecting commonly used groupings in the literature (Clausen et al., 2018; Sarnocinska et al., 2016). Kendall's rank (nonparametric) correlation tests were carried out between the detections on the C‐POD and F‐POD at the scale of each temporal detection metric and for each train quality classification. Detections under the HiModLo filter were subsequently used in further statistical analysis of spatiotemporal trends.

Both monthly and seasonal DPH per day (filter HiModLo) were summarized for both the C‐POD and the F‐POD and compared using a detection ratio, expressed as: CF = Det_F/Det_C. This ratio was used to explore the comparability between the PODs across time and by what margin the F‐POD detects more echolocation clicks than the C‐POD.

Data on echolocation clicks were also exported and used to identify foraging events. Porpoise foraging behavior can be identified based on the duration of the inter‐click interval (ICI), or time between successive echolocation clicks within detected click trains (Verfuß et al., 2009). Foraging buzzes are presumed prey capture attempts with a typical ICI of less than 10 ms (DeRuiter et al., 2009; Miller, 2010; Verfuß et al., 2009). Gaussian mixture models were used to categorize echolocation clicks based on their ICI (Berges et al., 2019; Pirotta, Brookes, et al., 2014; Pirotta, Thompson, et al., 2014; Todd et al., 2022). This method classifies log‐transformed ICIs to identify patterns of echolocation clicks. Distinct ICIs are clustered and identified as either regular echolocation clicks (for example for navigation or searching for prey), foraging buzzes, (a presumed prey capture attempt with an ICI of <10 ms), or an inter‐train ICI (representing pauses between click trains). For further analysis, a buzz rate was calculated as buzz‐positive minutes (BPM) per hour/DPM per hour × 100. This rate was calculated to give a reflection of the proportion of time harbor porpoise were detected foraging as a function of their occurrence within the same period.

### 2.3 Environmental data

Sunrise, sunset, and civil twilight times were extracted from (www.timeanddate.com/sun) for Sherkin Island and were used to calculate the diel cycle phases (morning, day, evening, and night) (Carlström, 2005; Todd et al., 2009). Tide data were extracted from tide tables for Roaringwater Bay (www.tides4fishing.com/ie/munster/roaringwater‐bay). Time difference to the nearest high tide was calculated, as well as phases of the tidal cycle (ebb/flow/high/low water). Tidal range was also calculated as an indicator of spring and neap tides. Hourly seawater temperature data were obtained from both PODs. As POD temperature can be considered relative, the temperature value used in the analysis was calculated as a mean of the hourly mean POD recorded temperature, recorded from both the C‐POD and the F‐POD to reduce any recording bias. While the PODs cannot provide a direct measure of environmental noise levels, the click detection algorithms record unfiltered short click‐like events within the 20–160 kHz bandwidth (*Nall*) and are recorded for each sampling minute by both pods. A positive correlation has been found between *Nall* and environmental noise levels using a full‐bandwidth recorder and is used as a proxy for general noise levels (Clausen et al., 2018; Nuuttila et al., 2018). We included this in models to investigate the effect of varying environmental noise levels on POD detection performance. For the purpose of interpretation of the results, seasons were defined as spring (March to May), summer (June to August), autumn (September to November), and winter (December to February).

### 2.4 Statistical modeling

Statistical analyses were undertaken using R version 4.1.2 (R Core Team, 2022). Prior to statistical modeling, data exploration was conducted following Zuur et al. (2010). Autocorrelation was observed in the data using ACF plots with *itasdug* package (van Rij et al., 2015). Generalized Additive Models (GAMs) were conducted using the function *bam* within the *mgcv* package, which is optimized to deal with large datasets. GAMs were fitted with autoregressive (AR(1)) correlation structure to account for observed autocorrelation, and a negative binomial error distribution (*theta* values obtained using function *gam* and *nb* distribution), with logarithmic link function, to deal with zero‐inflation in the data (Wood, 2011; Wood et al., 2015). The *rho* values for the AR structure (which control the degree of permitted autocorrelation; Wood, 2017) were determined using the *itsadug* package and ACF plots. The parameter gamma was set to 1.2 to reduce the potential overfitting of splines.

The data were analyzed for every hour and the response variables used were the number of minutes with porpoise detections for each hour (0–60 DPM) and the buzz rate ([BPM per hour/DPM per hour] × 100), as a measure of foraging activity. Explanatory variables included diel period as a factor and month, temperature, noise, difference to high tide, and tidal range as smooth terms. Circular smoothers were used for month and difference to high tide. Thin‐plate regression splines with shrinkage were used for the remaining smooth terms, which return the simplest effective spline. Generalized cross‐validation and manual knot selection were used, with chosen values visually selected based on the trade‐off between the overall simplicity of the model and the explanatory power of smooth graphs. To decide between the appropriate tidal variable for analysis, each was included in the full model and models compared based on AIC score. Time difference to high tide resulted in the model with the lowest AIC and was used for further analysis.

The relatedness between the smooth terms in the model was measured using the function *concurvity*, in a similar manner to variance inflation factors used for generalized linear models (GLM). Relatedness was measured on a scale of 0–1, with 0 indicating no difference and 1 indicating that terms are identifiable from each other. Concurvity was not found, so all terms were retained for analysis. Stepwise model selection was performed where nonsignificant interactions were dropped from the model (starting with the least significant) and model validation repeated. Models were compared using AIC to choose the best and final model. Model performance was checked using gam.check based on traditional QQ plot and residual plots (Wood, 2006). Model goodness of fit was described by deviance explained, and area under the receiver operator curve (AUC), package *caret* (Kuhn, 2022). AUC was calculated by predicting a binomial response variable from the fitted model and compared with the observed presence/ absence of the variable. This results in a value ranging from 0 to 1, with values closer to 1 indicating better model fit (Boyce et al., 2002). Graphical outputs were produced using the *mgcViz* package (Fasiolo et al., 2018) and *ggplot2* (Wickham, 2009).

## 3 RESULTS

### 3.1 Comparability in the detection of harbor porpoise

The C‐POD and F‐POD were co‐deployed for a continuous period of 444 days between April 2021 and July 2022. Harbor porpoise detections were recorded on 94% of days on the C‐POD (419 days), and 98% on the F‐POD (433 days) under all train quality filters.

**FIGURE 1:**
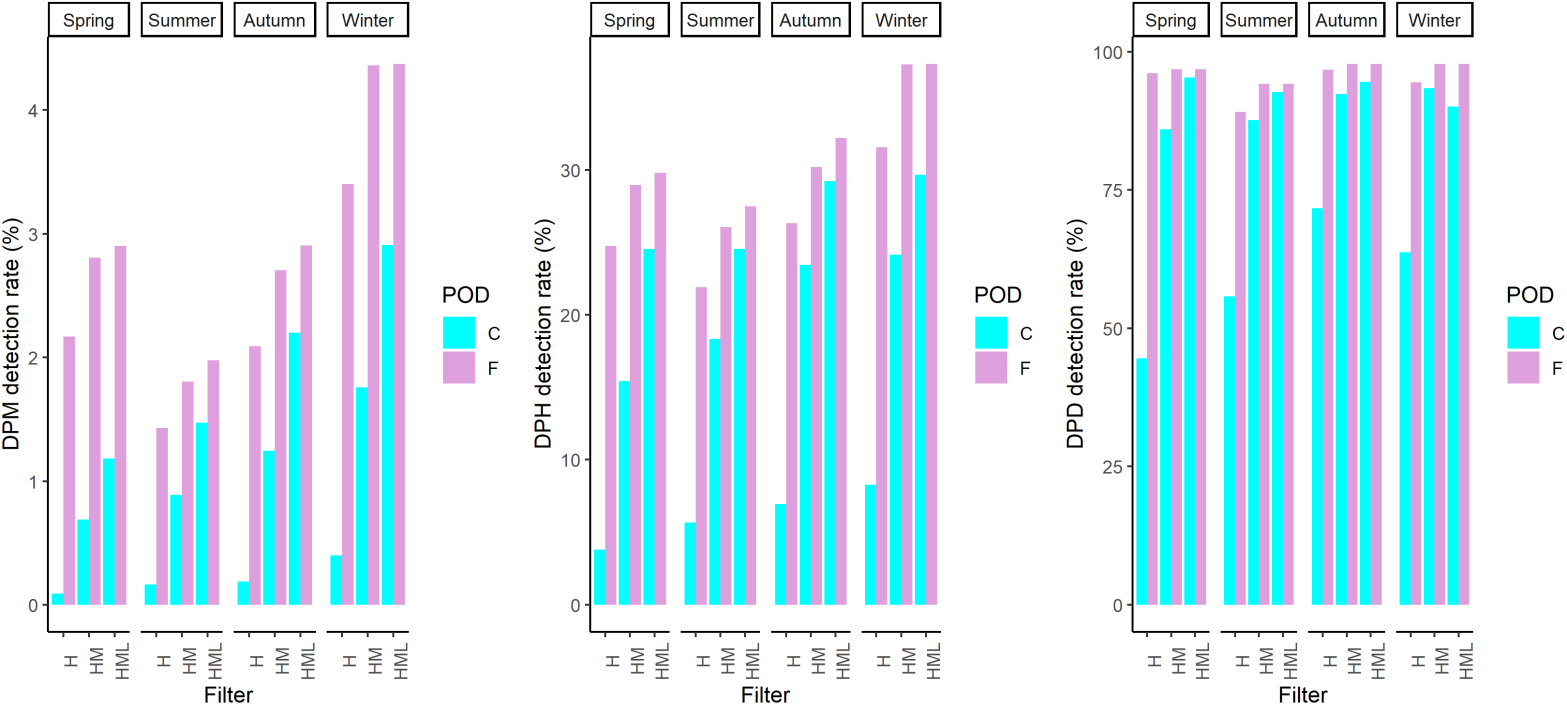
Detection‐positive minutes (DPM) detection rate (%), detection‐positive hours (DPH) detection rate (%), and detection‐positive days (DPD) detection rate (%) for C‐POD and F‐POD co‐deployment. Different PODs specified in the legend and collated per season across the deployment period. HML represents high‐, moderate‐, and low‐quality train classification categories. Detection rates were standardized to account for unequal recording effort between seasons, that is, DPM detection rate was calculated as follows: DPM/total number of minutes monitored per each season × 100. DPH and DPD rates calculated in an analogous manner but standardized to total number of hours/ days per season, respectively.

Across all deployments, total C‐POD detections were lower than those recorded using the F‐POD (Figure 1). The margin of difference was highly dependent on the detection metric and the train quality classification category examined, with smallest differences noted when combining all (visually validated) quality categories. False positive rates typically ranged from 1% to 5% for C‐POD HiMod detections, and lower for F‐POD HiMod detections at 0%–3%. The inclusion of low‐quality trains on average increased the false positive rate by 1%–2%. While there was a significant correlation between the detections on the co‐deployed PODs at all temporal scales, PODs were in greatest agreement at the broadest scale metric of DPD (filter HiMod *r* = .86, *p* < .05; HiModLo *r* = .84, *p* < .05, Online Appendix, Table S2, Figure 1), and least comparable at the scale of the number of clicks per hour (Online Appendix, Table S2). Although variable across time, the increased capacity for click detection by the F‐POD was evident, with the F‐POD detecting 10 times or more the number of clicks detected by the C‐POD (Online Appendix, Table S1). Considering the “Hi” filter alone there was limited comparability between the C‐POD and the F‐POD, with significant but weak correlation (Online Appendix, Table S2). Moreover, using only the high‐quality classification filter meant that on average 75% of the F‐POD DPH were not detected by the C‐POD within the same quality grouping. Using the HiMod or HiModLo groupings increased the comparability of PODs, but the F‐POD still consistently recorded more harbor porpoise detections overall (Figure 1). There was a small proportion of DPH recorded on the C‐POD that were not matched by the F‐POD. However, these visually validated C‐POD detections often matched unclassified NBHF clicks (i.e., not defined to be click trains by any of the quality classes) on the F‐POD. Furthermore, most unmatched C‐POD detections were weaker trains of Low‐quality, occurring during periods of increased ambient noise, and not classified by the more stringent F‐POD algorithms.

**FIGURE 2:**
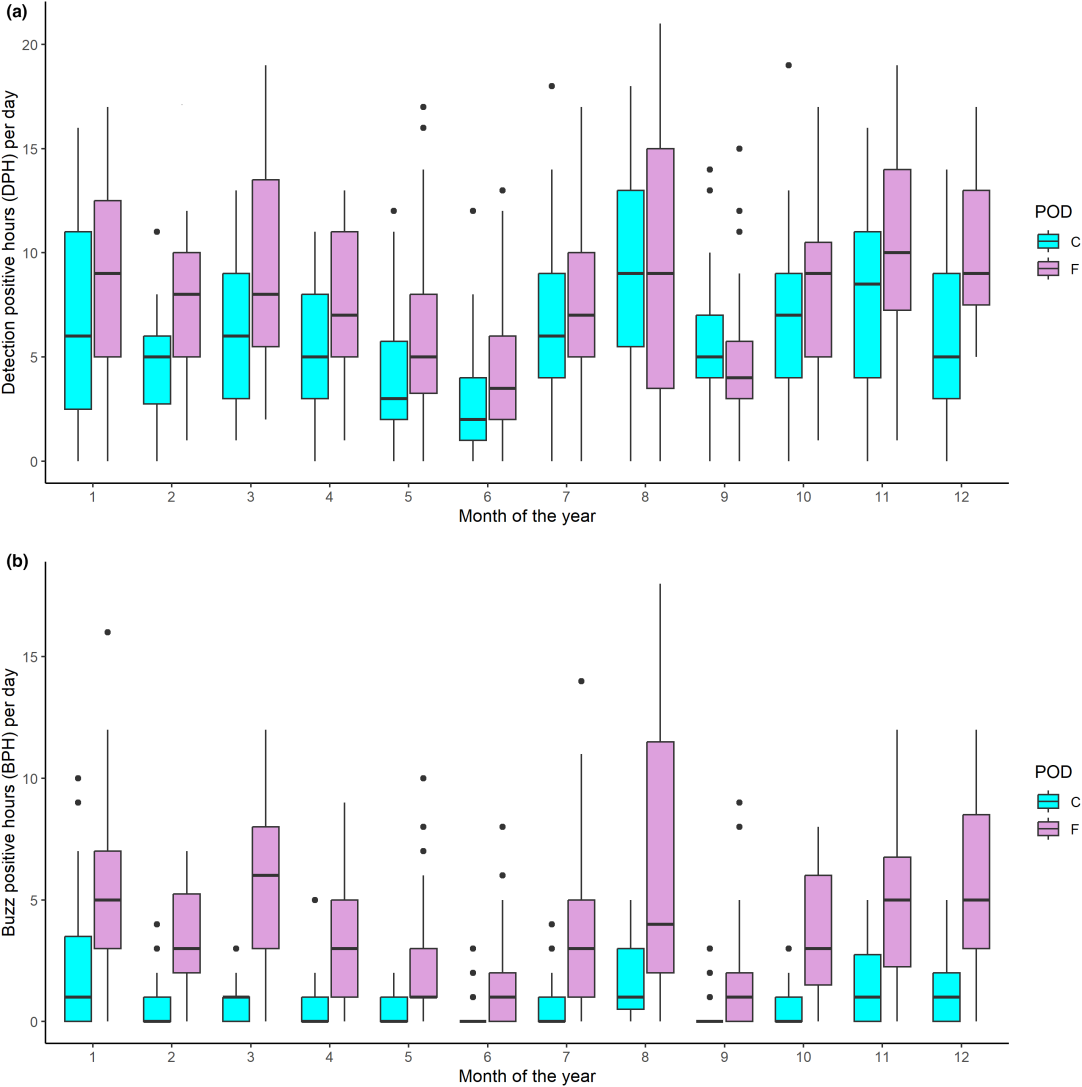
(a) Median monthly detections (detection‐positive hours per day [DPH]) for the C‐POD and F‐POD co‐deployment. (b) Median buzz‐positive hours (BPH, i.e., cumulative number of hours per day where at least one foraging buzz was detected). Different PODs specified in the legend. Boxplot shows the lower quartile, the median, and the upper quartile with the whiskers extending to the most extreme data points (1.5 times either side of the interquartile range).

Over a total of 15‐month period, the PODs were deployed and a detection ratio of 1.38 was calculated using DPH (filter HiModLo). Seasonal variability in this detection ratio occurred with PODs least comparable in the spring–summer when detection rates were lowest (ratios: spring: 1.52, summer: 1.48, autumn: 1.07, winter: 1.37). Despite the detection differences, both PODs similarly identified temporal patterns of occurrence at hourly scales (DPM and DPH) using the HiMod or HiModLo train quality groupings. Seasonal patterns are reflected with a decrease in detections from April to July and consistently more detections throughout the winter months (Figure 2).

Harbor porpoise foraging behavior was detected by both of the co‐deployed PODs throughout the deployment period. Foraging buzzes were found to account for approximately 8% of the total clicks recorded by the C‐POD, compared with 26% for the F‐POD. The number of buzz‐positive hours per day (BPH) was found to vary seasonally, reflecting the temporal patterns shown in overall harbor porpoise detections (Figure 2), with low counts of BPH from May till July, and peaks shown throughout winter, as well as for August (Figure 2).

### 3.2 Spatiotemporal drivers of harbor porpoise occurrence and foraging activity

GAMs were run to compare the temporal trends and environmental predictors of harbor porpoise occurrence between the C‐POD and F‐POD. The best models for DPM per hour (DPM/h) from both PODs retained all explanatory variables (all variables significant in both the C‐POD and the F‐POD models). Effect sizes of variables retained within the models were remarkably consistent between the C‐POD and F‐POD models, with the exception of noise, where this showed a considerably higher effect within the C‐POD model (Table 1).

**TABLE 1:** Final model summaries for harbor porpoise occurrence GAMs for both PODs.

Retained terms	C‐POD model		F‐POD model	
	*p*‐value	Effect size	*p*‐value	Effect size
Month	**<.001**	18.84 (++)	**<.001**	19.89 (++)
Temp (°C)	**.002**	6.07 (+)	**<.001**	3.61 (+)
Nall‐noise proxy	**<.001**	59.57 (+++)	**<.001**	2.90 (+)
Tidal range (m)	**.01**	6.53 (+)	**<.001**	3.75 (+)
Diff. to HT	**<.001**	2.91 (+)	**<.001**	1.26 (+)
Diel period (relative to day)				
Evening	.2048	−1.27 (−)	**<.001**	−3.98 (−)
Morning	.1271	−1.53 (−)	**.01**	−2.56 (−)
Night	**<.001**	−4.42 (−)	**<.001**	−7.23 (−)

*Note*: Significant interactions are indicated by *p*‐value <.05, and bold text. Effect size derived from *F* statistic for smooth terms, and *t* value for fitted coefficients. Strength and direction of the relationship (i.e., positive or negative) are indicated in brackets following the effect size (Full model output available in the Online Appendix, Table S3).

**FIGURE 3:**
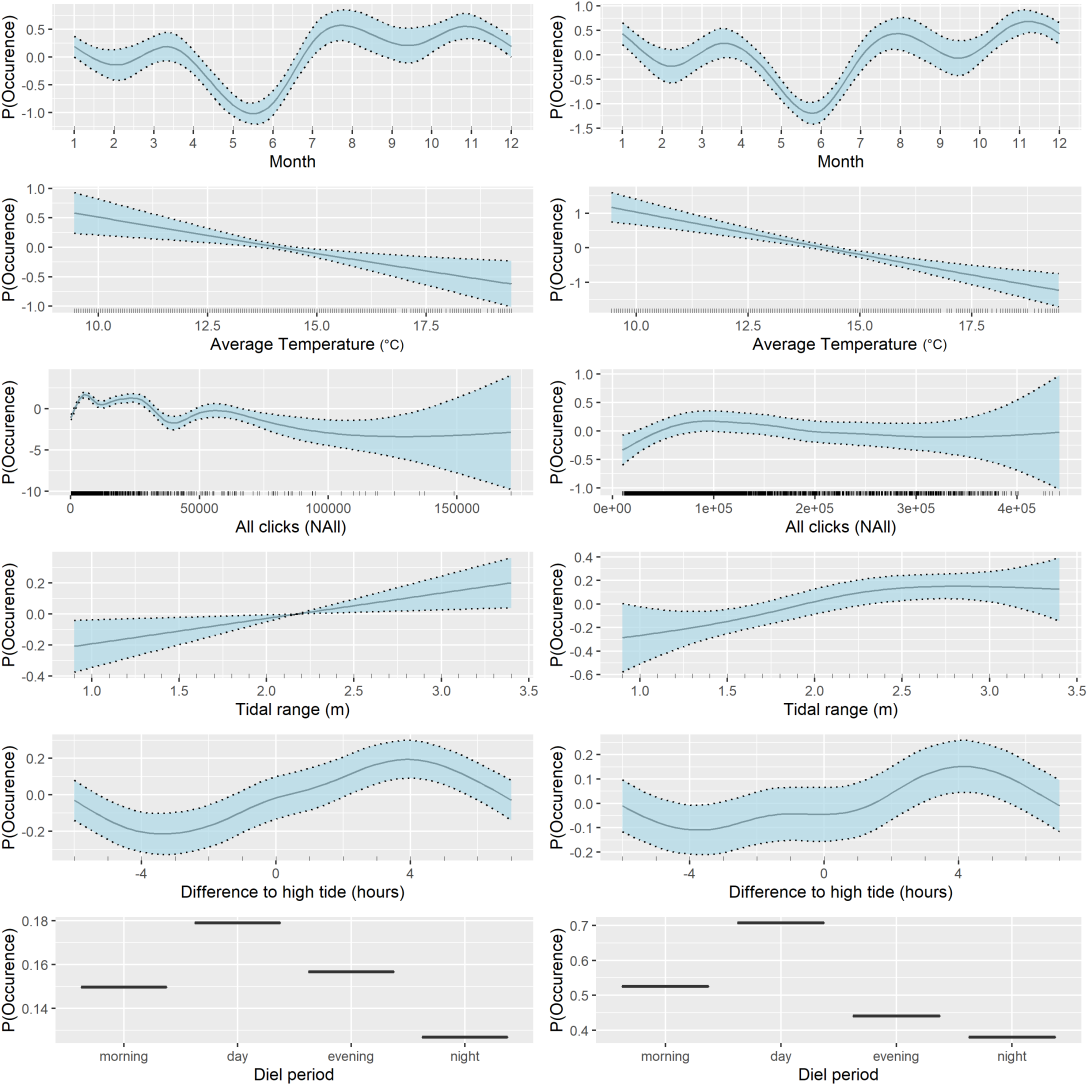
Significant relationships for the final C‐POD (left) and F‐POD (right) harbor porpoise detection model based on GAM/BAM standard errors. y axis demonstrates the probability of detecting harbor porpoise according to the explanatory variables demonstrated on the x axis. Shaded areas represent 95% confidence intervals.

Similar temporal trends were highlighted by the C‐POD and F‐POD models in terms of harbor porpoise DPM/h per month and throughout the diel cycle (Figure 3). Both tidal range and time to high tide were significant, reflecting similar trends from both devices with an increase in detections during the ebb tide and during tidal ranges associated with spring tides (Figure 3); however, both variables had relatively small effect sizes within the model (Table 1). The C‐POD model highlighted a much greater effect of noise on harbor porpoise detections, with a clear decrease in porpoise detections at higher noise levels, occurring at a much lower noise threshold for the C‐POD than the F‐POD (Figure 3). Harbor porpoise detections were found to decrease with increasing water temperature for both devices (Figure 3).

In contrast to the similar temporal patterns shown via the occurrence, strong differences occurred between models of foraging activity between C‐PODS and F‐PODs using buzz rate per hour as the response variable.

**FIGURE 4:**
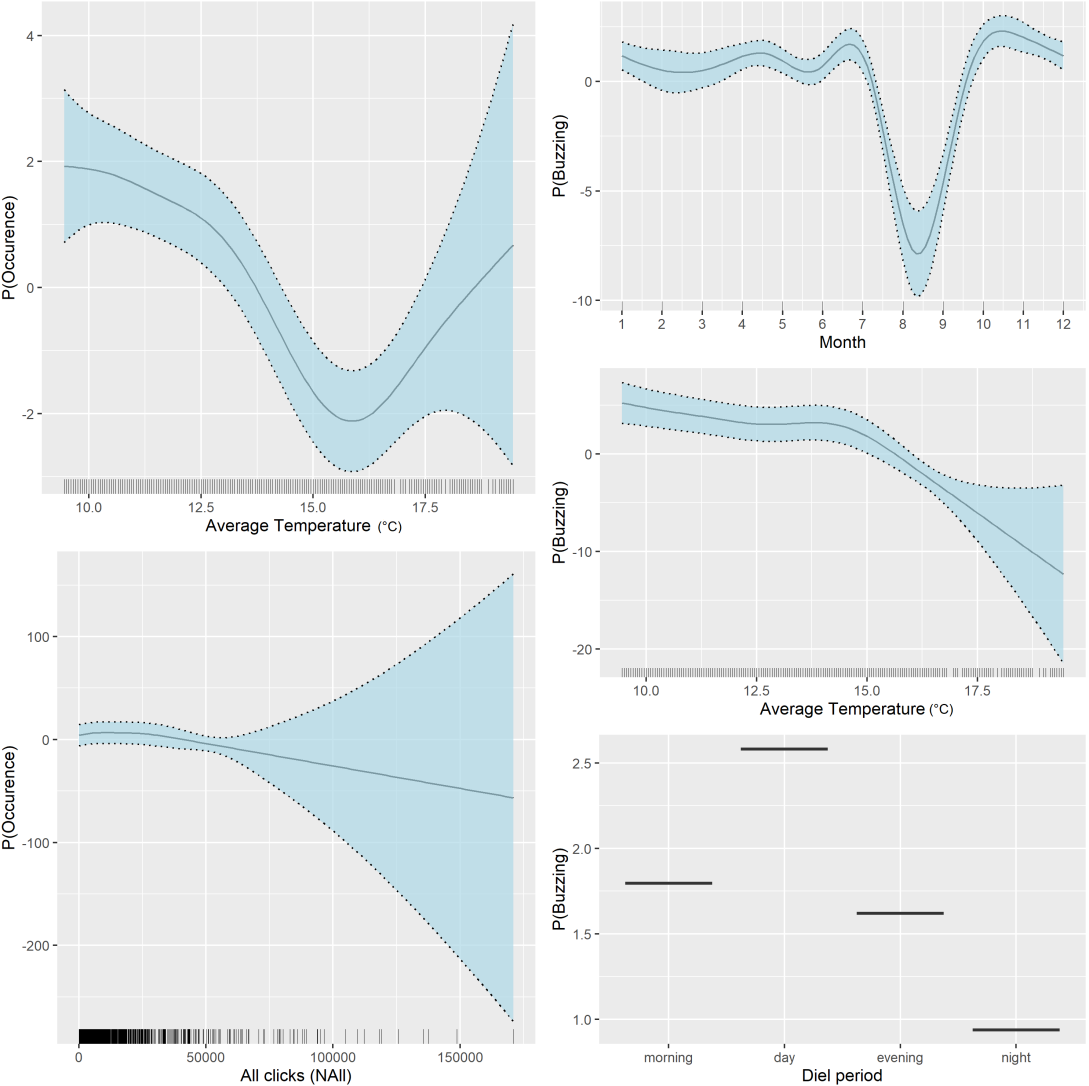
Significant relationships for the final C‐POD (left) and F‐POD (right) harbor porpoise foraging model based on GAM/BAM standard errors. y axis demonstrates the probability of harbor porpoise foraging activity according to the explanatory variables demonstrated on the x axis. Shaded areas representing 95% confidence intervals.

The C‐POD foraging model did not retain any temporal variables despite them remaining the most influential covariates within the F‐POD foraging model. Neither time difference to high tide nor tidal range was found to influence harbor porpoise foraging behavior in either C‐POD or F‐POD models. The F‐POD model suggested a decrease in foraging rate between July and September, and an increase in foraging rate detected during the daytime (Figure 4). Similar to the detection model, foraging activity decreased with increasing water temperature, with highest buzz detections around 10°C. An increase in foraging rate was also observed from the C‐POD only in anomalous water temperatures of around 17.5°C (Figure 4), these instances were investigated and found to be during a heatwave in July 2021. In contrast to the C‐POD model, the F‐POD foraging model found no significant effect of noise on foraging activity (Table 2).

**TABLE 2:** Final model summaries for foraging harbor porpoise GAMs for both PODs.

Retained terms	C‐POD model		F‐POD model	
	*p*‐value	Effect size	*p*‐value	Effect size
Month	.93	0.33	**<.001**	11.02 (++)
Temp (°C)	**<.001**	6.84 (+)	**<.001**	3.92 (+)
Nall‐noise proxy	**<.001**	5.06 (+)		
Tidal range (m)	.37	1.17		
Diff. to HT				
Diel period (relative to day)				
Evening	.30	−1.04 (−)	.12	−1.58 (−)
Morning	.88	−0.16 (−)	.19	−1.319 (−)
Night	.24	−1.168 (−)	**<.001**	−5.79 (−)

*Note*: Significant interactions are indicated by *p*‐value <.05, and bold text. Effect size derived from *F* statistic for smooth terms, and *t* value for fitted coefficients. Strength and direction of the relationship (i.e., positive or negative) are indicated in brackets following the effect size (Full model output available in the Online Appendix, Table S4).

## 4 DISCUSSION

To the best of our knowledge, this study is the first to evaluate the performance of co‐deployed C‐POD and F‐POD devices in a field setting for monitoring harbor porpoise. Although this study occurred in one location and focused on one species, it is an important and timely starting point for considering the transition from C‐PODs to F‐PODs. Previous studies have evaluated the performance of the C‐POD with other types of full‐bandwidth recorders (including Soundtrap, DMON), with the C‐POD typically performing well with an overall high degree of accuracy (Jacobson et al., 2017; Roberts & Read, 2015; Sarnocinska et al., 2016). Our results suggest that at appropriate temporal scales and with certain caveats, F‐PODs provide comparable results to the older C‐PODs and would be suitable replacements for C‐PODs in existing monitoring programs as C‐PODs reach the end of their serviceable lifetime.

Data from click detectors such as C‐PODs, its predecessor T‐PODs, and now the F‐PODs have been used to monitor small cetaceans, as well as their responses to anthropogenic activities in numerous settings including pile driving, seismic surveys, and fisheries deterrent devices (e.g., Omeyer et al., 2020; Philpott et al., 2007; Thompson et al., 2013; Todd et al., 2020). Our results show that the F‐POD consistently detects more echolocation clicks and foraging buzzes than the C‐POD across the temporal scales of minutes, hours, and days, as well as all train quality classification groupings. Lower detection rates by the C‐POD are to be expected due to advances in F‐POD electronics and software to capture more information on individual echolocation clicks and enhance train detection (Chelonia Ltd, 2022). This poses a potential issue for researchers engaged in long‐term monitoring, with questions about how comparable different POD types may be, potentially affecting time series as C‐PODs are eventually replaced by F‐PODs. This study shows that both C‐PODs and F‐PODs detected similar patterns of occurrence and echolocation activity. As indicated by Garrod et al. (2018), detection metrics at a minimum of an hourly scale are representative of relative occurrence, enabling temporal trends to be determined. Detections at the broader scale of DPD were found to match best between both PODs with little discrepancy between them. Therefore, for direct comparison between C‐POD and F‐POD data, DPD, and using combined classifications of Hi‐Mod and Lo, is the only detection metric recommended. However, such a metric would be insufficient for identifying fine‐scale temporal patterns of occurrence or behavior in response to factors such as diurnal changes in prey availability or tidal state, both known to influence harbor porpoise occurrence and feeding behavior (Schaffeld et al., 2016; Zein et al., 2019). Furthermore, in low‐density areas, detections even at the broad scale of DPD may be insufficient in identifying seasonal patterns.

Our results highlighted that F‐PODs appear to be much more capable at identifying harbor porpoise click trains with confidence than the C‐POD (i.e., classified as high quality by the KERNO classifier). However, when considering the combined train quality classification groupings (i.e., HiMod and HiModLo) the two PODs are substantially more comparable. Researchers considering using a time series consisting of data from C‐PODs and F‐PODs for analysis of temporal trends should consider using the combined classifications, provided extensive visual validation is followed, particularly for low‐quality trains to eliminate possible false positive detections. Areas with particularly high densities, where thorough validation of low‐quality trains may not be feasible, should consider only including HiMod data for analysis. The enhanced train detection specified by the manufacturer has also been demonstrated in our results with the F‐POD continually recording more harbor porpoise detections than the C‐POD by a factor of 1.38 across the deployment period even considering all train quality classes (i.e., HiModLo grouping). Comparability between the PODs was, however, found to be variable across seasons, with the highest detection ratio in spring and summer making detection rates on the PODs less comparable. Detection ratios such as those explored here could be investigated further within monitoring programs looking to transition to the use of F‐PODs. Detections from C‐PODs have been reported to vary with environmental conditions, such as depth (Sostres Alonso & Nuuttila, 2015), so it would be unsurprising for F‐POD detections to also vary across different deployment locations. We suggest that researchers should conduct regional comparisons before transitioning to F‐PODs entirely rather than adopting detection ratios from other regions as a correction factor. Additionally, should sufficient C‐POD and F‐POD comparisons take place across a range of locations, this could increase the potential to create a more universal detection ratio to evaluate the F‐POD performance to its C‐POD predecessor. Understanding how the devices compare in various deployment sites can help for the interpretation of long‐term data beyond the lifespan of the C‐POD and avoid misinterpretation of the data (for example interpreting a false increase in occurrence due to differing device sensitivities). Such a misinterpretation of increased occurrence using F‐PODs could be a considerable problem for large‐scale monitoring programs for vulnerable populations, such as the Baltic harbor porpoise with the SAMBAH project using over 300 C‐PODs for trend assessments (Amundin et al., 2022; SAMBAH, 2016). C‐PODs in this region have been successful in identifying a yearly increase in the porpoise population of 2.4% from 2011 to 2019 (Owen et al., 2021). In many regions fine‐scale trends are important and comparing detection rates derived from F‐PODs to existing C‐POD detection rates could negatively influence conservation measures if not appropriately interpreted.

Investigating spatial and temporal patterns in species occurrence is often the crux of ecological monitoring (e.g., Jones et al., 2014; Williamson et al., 2017; Zein et al., 2019). Generalized additive models were used to investigate whether detections from both types of POD have the capacity to identify the same temporal drivers of porpoise occurrence and foraging activity. The occurrence models for both PODs highlighted the same temporal patterns, and same environmental predictors with very similar effect sizes, suggesting that analyses using data from both C‐PODs and F‐PODs will not be affected greatly by POD type. However, it would be prudent to include POD type as a fixed factor in any such analysis. Conversely, the models investigating foraging rates did not provide analogous results. No temporal patterns were found using the C‐POD models; however, the overall number of clicks detected by the C‐POD and subsequently the number of foraging buzzes identified was considerably less than those identified by the F‐POD (Online Appendix, Table S1). The higher buzz rates from the F‐POD enabled the detection of temporal patterns including an increase in the foraging rate from autumn to winter and an increase in foraging activity during the day. The specific nature of the relationships and their ecological context is outside of the scope of the current study, but the contrasting ability of the PODs to detect feeding rates is particularly relevant in the context of integrating F‐PODS into long‐term datasets. The increased click detection capacity of the F‐POD now enables fine‐scale analysis of foraging or social behaviors (demonstrated by high click rates; Clausen et al., 2010) that have perhaps been missed or underestimated using C‐PODs. As buzz rates in the current study were relatively low, investigating this matter in a high occupancy area or known porpoise foraging area would prove valuable to understanding more about these echolocation behaviors. Additionally, F‐PODs were found to be less affected by environmental noise levels within the 20‐160KHz noise band, as indicated by *Nall* (Clausen et al., 2018). It is plausible that decreased detections on the C‐POD during periods of increasing environmental noise and sea water temperature fluctuations are a consequence of detector performance, which has been overcome during the development of the F‐POD in conjunction with the increased click detection ability.

Long‐term datasets and consistency of monitoring methods throughout the duration of monitoring programs are important to enable long‐term trends to be identified, particularly in areas of high conservation importance. Changing controllable factors such as monitoring equipment can skew our understanding of these long‐term trends and in turn make it more difficult to interpret a change in habitat use, or behavior of a species, which can be detrimental in the event of a disturbance or imminent threat.

Static acoustic monitoring using PODs has been an integral part of cetacean monitoring programs exploring habitat use and behavior. Our results show that the C‐POD and the F‐POD are consistently comparable at the broad scale of identifying porpoise presence, and produce similar results when modeling environmental correlates of occurrence. However, the C‐POD failed to detect the more nuanced patterns detected by the F‐POD, particularly when investigating foraging behavior versus occurrence. On account of its greater sensitivity and increased detection rates for harbor porpoise the F‐POD certainly can be a useful tool to integrate into acoustic monitoring programs. While the introduction of F‐PODs into long‐term time series is unlikely to change our understanding of the environmental drivers of occurrence, it is advisable that detections from one POD type should not be directly compared with detections from another as this could give erroneous results of increased occurrence due to differing detection rates rather than a true increase in individuals. Furthermore, any studies transitioning between PODs or combining C‐POD and F‐POD data should consider including POD type as a factor when conducting time‐series analyses. While the current study only investigated comparability in POD performance for detecting the acoustic activity of harbor porpoise, it is likely that analogous differences would be seen for other cetacean species recorded by PODs. While this study only considered a comparison between a co‐deployed C‐POD and F‐POD at a single monitoring location, it has provided important and timely insights into the integration of the F‐PODs into existing C‐POD monitoring programs or as a comparison of F‐POD detection rates with other studies. We urge other POD users to consider undertaking similar comparisons in other regions to assess the comparability of C‐POD and F‐POD detections, simultaneously gathering data that could be aggregated and analyzed to develop a standardized correction factor in the future.

## AUTHOR CONTRIBUTIONS

**Nicole Rose Eileen Todd:** Conceptualization (equal); data curation (equal); formal analysis (equal); funding acquisition (equal); methodology (equal); project administration (equal); writing – original draft (lead); writing – review and editing (equal). **Ailbhe Sarah Kavanagh:** Conceptualization (equal); supervision (equal); writing – review and editing (equal). **Emer Rogan:** Conceptualization (equal); supervision (equal); writing – review and editing (equal). **Mark John Jessopp:** Conceptualization (equal); supervision (equal); writing – review and editing (equal).

## CONFLICT OF INTEREST STATEMENT

The authors declare no conflict of interest.

### OPEN RESEARCH BADGES

This article has earned an Open Data badge for making publicly available the digitally‐shareable data necessary to reproduce the reported results. The data is available at https://github.com/Ntodd95/What_the_F‐POD.

## Supporting information


Appendix S1.

## Data Availability

All processed data and R code files to reproduce the results given in this paper are available in the following repository: https://zenodo.org/badge/latestdoi/639346339.
