# Supplementary material for: What the F‐POD? Comparing the F‐POD and C‐POD for monitoring of harbor porpoise (Phocoena phocoena)
Source: Ecol Evol. 2023 Jun 9;13(6):e10186. doi: 10.1002/ece3.10186 (PMC10256617; doi:10.1002/ece3.10186)
Supplement: Supplementary file 1 — Appendix S1. [file ECE3-13-e10186-s001.docx]

Comparing the F-POD and C-POD for monitoring of harbour porpoise

Supplementary material


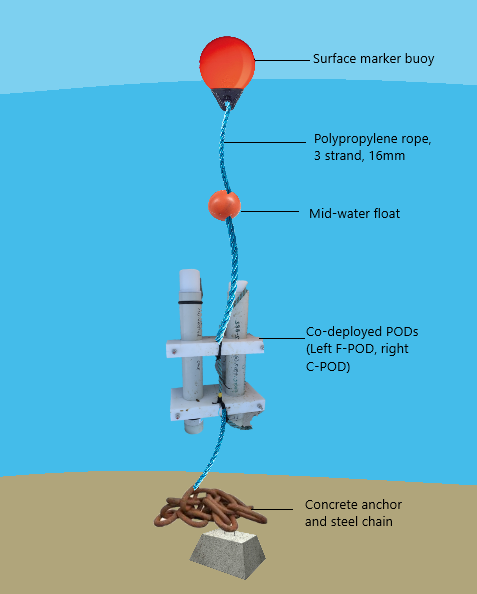


Figure S1: Diagram of mooring design of the co-deployed C-POD and F-POD. PODs are side aligned in a custom-made acetal plastic frame constructed for optimisation of simultaneous detections.

Table S1: Summary of detection metrics (DPM = Detection positive minutes, DPH = Detection positive hours, DPD = Detection positive days) for C-POD and F-POD co-deployment across all deployment periods. HML represents High, Moderate, and Low-quality train classification categories. Total number of deployment days specified per each deployment period.

|  |  | **Dep 1 (27/4/21-14/07/21)**  **Total days = 79** | | **Dep 2 (14/07/21- 09/11/21)**  **Total days = 118** | | **Dep 3 (09/11/21- 24/03/22)**  **Total days= 135** | | **Dep 4 (24/03/22-14/07/22)**  **Total days= 113** | |
| --- | --- | --- | --- | --- | --- | --- | --- | --- | --- |
|  |  | **C** | **F** | **C** | **F** | **C** | **F** | **C** | **F** |
| **HML** | **NClx** | 21614 | 218832 | 101151 | 564646 | 165249 | 1192186 | 16156 | 248817 |
|  | **DPM** | 946 | 2325 | 4115 | 4930 | 5855 | 9313 | 913 | 2288 |
|  | **DPH** | 360 | 514 | 941 | 998 | 1008 | 1241 | 402 | 551 |
|  | **DPD** | 74 | 79 | 114 | 116 | 126 | 134 | 105 | 104 |
| **HM** | **NClx** | 12831 | 209274 | 58827 | 536528 | 96707 | 1111788 | 8284 | 248817 |
|  | **DPM** | 555 | 2050 | 2404 | 4461 | 3519 | 9277 | 510 | 2273 |
|  | **DPH** | 257 | 514 | 757 | 933 | 824 | 1240 | 228 | 547 |
|  | **DPD** | 70 | 79 | 109 | 116 | 129 | 134 | 92 | 104 |
| **H** | **NClx** | 4363 | 166033 | 15785 | 400035 | 28702 | 796291 | 1480 | 158708 |
|  | **DPM** | 111 | 1535 | 393 | 3521 | 704 | 7291 | 52 | 1722 |
|  | **DPH** | 69 | 426 | 239 | 810 | 284 | 1071 | 47 | 447 |
|  | **DPD** | 42 | 77 | 89 | 114 | 96 | 131 | 31 | 69 |

Correlation coefficients:

Table S2: Kendall’s Tau correlation coefficient reported for DPM, DPH (per deployment) and DPD (pooled for all deployments). All coefficients significant (i.e. p<0.05). HML represents High, Moderate, and Low-quality train classification categories.

| Filter | Dep 3 | | | Dep 4 | | | Dep 5 | | | Dep 6 | | | All dep |
| --- | --- | --- | --- | --- | --- | --- | --- | --- | --- | --- | --- | --- | --- |
|  | **DPM** | **DPH** | **NClx** | **DPM** | **DPH** | **NClx** | **DPM** | **DPH** | **NClx** | **DPM** | **DPH** | **NClx** | **DPD** |
| HML | 0.65 | 0.68 | 0.64 | 0.72 | 0.76 | 0.30 | 0.37 | 0.63 | 0.48 | 0.55 | 0.63 | 0.49 | 0.84 |
| HM | 0.6 | 0.64 | 0.59 | 0.7 | 0.72 | 0.31 | 0.35 | 0.67 | 0.46 | 0.50 | 0.59 | 0.49 | 0.86 |
| H | 0.365 | 0.4 | 0.36 | 0.47 | 0.63 | 0.21 | 0.23 | 0.54 | 0.29 | 0.19 | 0.26 | 0.19 | 0.5 |

Full GAM model outputs:

Table S3: Final model summaries for harbour porpoise occurrence for both PODs. For smooth terms included in the GAMs, estimated degrees of freedom (edf) is a measure of complexity, F-statistic is a measure of each covariates effect on the model output. For parametric coefficients, Positive estimates indicate a positive relationship between the predictor and the response variable, whereas negative estimates indicate a negative relationship. Standard error is presented, and the *T*-value is analogous to the *F*-statistic for smoothed terms. Significant interactions are indicated by *p*-value <0.05, and bold text.

| Retained terms | Edf (for smooth terms) | df | F-statistic | p-value | Estimate (fitted coefficients) | Std. Error (fitted coefficients) | T-value | % Dev. Expl. | Adj. R |
| --- | --- | --- | --- | --- | --- | --- | --- | --- | --- |
|  | **Harbour porpoise occurrence model (C-POD)** | | | | |  |  | 23.8 | 0.08 |
| Month | 6.94 | 8.00 | 18.84 | **<0.001** |  |  |  |  |  |
| Temp | 1.74 | 2.23 | 6.07 | **0.002** |  |  |  |  |  |
| Nall- All clicks | 8.49 | 8.89 | 59.57 | **<0.001** |  |  |  |  |  |
| Tidal range | 1 | 1 | 6.53 | **0.01** |  |  |  |  |  |
| Diff. to HT |  | 8.00 | 2.91 | **<0.001** |  |  |  |  |  |
| Diel period  (Relative to Day) | - | - | - | Evening:  0.20  Morning:  0.12  Night:  **<0.001** | Evening:  -0.15  Morning:  -0.18  Night:  -0.34 | Evening:  0.12  Morning:  0.12  Night:  0.08 | Evening:  -1.27  Morning:  -1.53  Night:  -4.41 |  |  |
|  |  |  |  |  |  |  |  |  |  |
|  | **Harbour porpoise occurrence model (F-POD)** | | | | |  |  | 11.8 | 0.07 |
| Month | 6.8822 | 8.00 | 19.890 | **<0.001** |  |  |  |  |  |
| Temp | 1.1126 | 9.00 | 3.607 | **<0.001** |  |  |  |  |  |
| Nall- All clicks | 3.6644 | 4.617 | 2.901 | **<0.001** |  |  |  |  |  |
| Tidal range | 1.917 | 2.434 | 3.750 | **<0.001** |  |  |  |  |  |
| Diff to HT | 2.616 | 8.00 | 1.263 | **<0.001** |  |  |  |  |  |
| Diel period (relative to Day) | - | - | - | Evening:  **<0.001**  Morning:  **0.01**  Night:  **<0.001** | Evening:  -0.47385  Morning:  -0.29732  Night:  -0.62258 | Evening:  0.11899  Morning:  0.11608  Night:  0.08611 | Evening:  -3.982  Morning:  -2.561  Night:  -7.230 |  |  |

| Retained terms | Edf (for smooth terms) | df | F-statistic | p-value | Estimate (fitted coefficients) | Std. Error (fitted coefficients) | T-value | % Dev. Expl. | Adj. R |
| --- | --- | --- | --- | --- | --- | --- | --- | --- | --- |
|  | **Harbour porpoise foraging model (C-POD)** | | | | |  |  | 12.2 | -0.002 |
| Month | 0.93 | 8.00 | 0.33 | 0.051 |  |  |  |  |  |
| Temp | 5.01 | 9.00 | 6.84 | **<0.001** |  |  |  |  |  |
| Nall- All clicks | 3.38 | 4.13 | 5.06 | **<0.001** |  |  |  |  |  |
| Tidal range | 1.99 | 2.51 | 1.14 | 0.37 |  |  |  |  |  |
| Diel period (relative to Day) | - | - | - | Evening:  0.30  Morning:  0.88  Night:  0.24 | Evening:  -0.58  Morning:  0.08  Night:  0.35 | Evening:  0.56  Morning:  0.53  Night:  0.30 | Evening:  -1.04  Morning:  -0.16  Night:  -1.168 |  |  |
|  |  |  |  |  |  |  |  |  |  |
|  | **Harbour porpoise foraging model (F-POD)** | | | | |  |  | 17.9 | -0.02 |
| Month | 7.72 | 8.00 | 11.02 | **<0.001** |  |  |  |  |  |
| Temp | 4.13 | 9.00 | 3.92 | **<0.001** |  |  |  |  |  |
| Diel period (relative to Day) | - | - | - | Evening:  0.12  Morning:  0.19  Night:  **<0.001** | Evening:  -0.47  Morning:  -0.38  Night:  -1.06 | Evening:  0.30  Morning:  0.29  Night:  0.18 | Evening:  -1.58  Morning:  -1.319  Night:  -5.79 |  |  |

Table S4: Final model summaries for harbour porpoise foraging GAMs for both PODs. Response is buzz rate ((BPM/DPM)*100) per hour for both models. For smooth terms included in the models, estimated degrees of freedom (edf) is a measure of complexity, F-statistic is a measure of each covariates effect on the model output. For parametric coefficients, Positive estimates indicate a positive relationship between the predictor and the response variable, whereas negative estimates indicate a negative relationship. Standard error is presented, and the *T*-value is analogous to the *F*-statistic for smoothed terms. Significant interactions are indicated by *p*-value <0.05, and bold text.
